# Sublobectomy and lymph node sampling are adequate for patients with invasive lung adenocarcinoma presenting as pure ground glass nodules

**DOI:** 10.1111/crj.13766

**Published:** 2024-05-07

**Authors:** Hansheng Wu, Junhan Wu, Xi Chen, Zihua Lan, Qibin Chen, Liangli Hong, Jinhai Yan, Shujie Huang, Jianrong Chen, Xirui Lin, Yong Tang, Haijie Xu, Guibin Qiao

**Affiliations:** ^1^ Department of Thoracic Surgery The First Affiliated Hospital of Shantou University Medical College Shantou China; ^2^ Shantou University Medical College Shantou China; ^3^ Department of Thoracic Surgery, Guangdong Provincial People's Hospital (Guangdong Academy of Medical Sciences) Southern Medical University Guangzhou China; ^4^ Department of Ultrasound Sichuan Provincial Maternity and Child Health Care Hospital Sichuan China; ^5^ Department of Pathology The First Affiliated Hospital of Shantou University Medical College Shantou China; ^6^ Department of Pathology, Guangdong Provincial People's Hospital (Guangdong Academy of Medical Sciences) Southern Medical University Guangzhou China

**Keywords:** lobectomy, lung adenocarcinoma, lymph node dissection, lymph node sampling, pure ground glass nodules, sublobectomy

## Abstract

**Purpose:**

In this study, we aimed to investigate the prognosis of invasive lung adenocarcinoma that manifests as pure ground glass nodules (pGGNs) and confirm the effectiveness of sublobectomy and lymph node sampling in patients with pGGN‐featured invasive adenocarcinoma (IAC).

**Materials and methods:**

We retrospectively enrolled 139 patients with pGGN‐featured IAC, who underwent complete resection in two medical institutions between January 2011 and May 2022. Stratification analysis was conducted to ensure balanced baseline characteristics among the patients. The 5‐year overall survival (OS) and disease‐free survival (DFS) rates were compared between the groups using Kaplan–Meier survival curves and log‐rank test.

**Results:**

The 5‐year OS and DFS rates for patients with IAC presenting as pGGNs after surgery were 96.5% and 100%, respectively. No lymph node metastasis or recurrence was observed in any of the enrolled patients. There was no statistically significant difference in the 5‐year OS between patients who underwent lobectomy or sublobectomy, along with lymph node resection or sampling.

**Conclusion:**

IAC presented as pGGNs exhibited low‐grade malignancy and had a relatively good prognosis. Therefore, these patients may be treated with sublobectomy and lymph node sampling.

## Graphical abstract

The findings of our study indicate that patients with invasive lung adenocarcinoma presenting as pure ground glass nodules exhibit a favorable 5‐year overall survival rate of 96.5%. No lymph node metastasis and recurrence were observed in all the enrolled patients. Our results also suggest that sublobectomy and lymph node sampling may be sufficient for managing patients with this particular presentation of invasive lung adenocarcinoma.

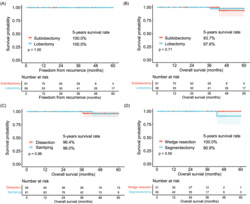

## 1 INTRODUCTION

Lung cancer remains the leading cause of cancer‐related death worldwide.^1^, ^2^ The popularization of high‐resolution computed tomography (CT) in the last decade, especially its extensive use as a diagnostic tool during the COVID‐19 pandemic, has led to a significant increase in the detection rate of lung ground glass nodules (GGNs).^2^, ^3^ Based on the proportion of solid components, GGNs can be categorized into mixed GGNs and pure GGNs (pGGNs).^4^ Pure GGNs are typically associated with focal interstitial fibrosis, atypical adenomatous hyperplasia, and adenocarcinoma in situ (AIS).^5^ However, several pieces of evidence still indicate that pGGNs have an incidence rate of 20%–40% for being minimally invasive adenocarcinoma (MIA) or invasive adenocarcinoma (IAC).^6^ Generally, IAC has a 5‐year disease‐free survival (DFS) ranging from 38% to 95% across different histological subtypes, while the 10‐year DFS rates of AIS and MIA are almost 100% after surgical resection.^7^

For early‐stage non‐small cell lung cancer (NSCLC), surgical resection is the dominant treatment modality.^8^ A randomized controlled trial in 1995 (LCSG821) demonstrated the prognostic superiority of lobectomy compared to sublobectomy, leading lobectomy to become the standard treatment for early‐stage NSCLC.^8^, ^9^ However, with recent advancements in imaging techniques, NSCLC detection has shown a tendency to occur at earlier stages and smaller sizes.^10^ Consequently, controversies surrounding the optimal surgical approach for early‐stage NSCLC are increasing.^11^, ^12^, ^13^ Among all the studies comparing surgical approaches, a series of research conducted by the Japanese Clinical Oncology Group has received significant scholarly attention. The results of these studies indicate that sublobectomy can achieve comparable overall survival (OS) and DFS in early‐stage NSCLC when there is no lymph node involvement and sufficient surgical margins can be achieved.^14^, ^15^ However, the proportion of pGGNs among pulmonary nodules is relatively low, and even fewer of them are diagnosed as invasive diseases.

Thus, patients with pGGN‐featured IAC constitute a distinct cohort that has been relatively underrepresented in previous research.^6^ To the best of our knowledge, there are only a few studies that specifically investigate the effectiveness of sublobectomy and lymph node sampling in IAC cases presenting as pGGNs.

To address this knowledge gap, we aimed to investigate the clinical outcomes of patients with IAC presented as pGGNs. We compared the prognosis of patients who received lobectomy, segmentectomy, and wedge resection. Additionally, we also compared the prognoses of patients undergoing lymph node dissection and lymph node sampling.

## 2 METHODS

### 2.1 Study population

This study included 139 patients with lung adenocarcinoma who underwent thoracoscopic lobectomy or sublobectomy at Guangdong Provincial People's Hospital and The First Affiliated Hospital of Shantou University Medical College between January 2011 and May 2022. The inclusion criteria were as follows: (1) High‐resolution CT (HRCT) revealed pGGNs before surgery and (2) pathologically diagnosed as IAC after surgery. The excluded criteria included (1) previous or concurrent other malignancy; (2) patients with multiple lesions; (3) patients who received chemotherapy or radiotherapy after surgery; and (4) patients with lymph node metastasis or distant metastasis found before surgery. Demographic and clinicopathological data of patients were obtained from the hospital's medical records. All the samples and data were collected after obtaining informed consent from each patient. This research was approved by the Ethics Committee of Guangdong Provincial People's Hospital (GDREC2019687H) and The First Affiliated Hospital of Shantou University Medical College (B‐2022‐149). This study was conducted following the guidelines of the Declaration of Helsinki (as revised in 2013).

### 2.2 Radiological and histopathological evaluation

In the present study, GGN is defined as a nodule with slightly increased density than the pulmonary parenchyma.^16^ GGNs were categorized as pGGNs if no consolidation component was detected on both the lung window and the mediastinal window in HRCT images with a layer thickness of 1 mm or lower.^16^, ^17^, ^18^ The consolidation component was defined as an area completely obscuring the underlying bronchovascular structures.^16^, ^18^ To identify these components, the lung window was set with a window width of 1500 HU and a window level of −400 HU, while the mediastinal window had a window width of 350 HU and a window level of 50 HU.^16^, ^19^ The imaging results were independently reviewed by a radiologist (Jianfa Zhang) and two thoracic surgeons (Guibin Qiao and Hansheng Wu).

**FIGURE 1:**
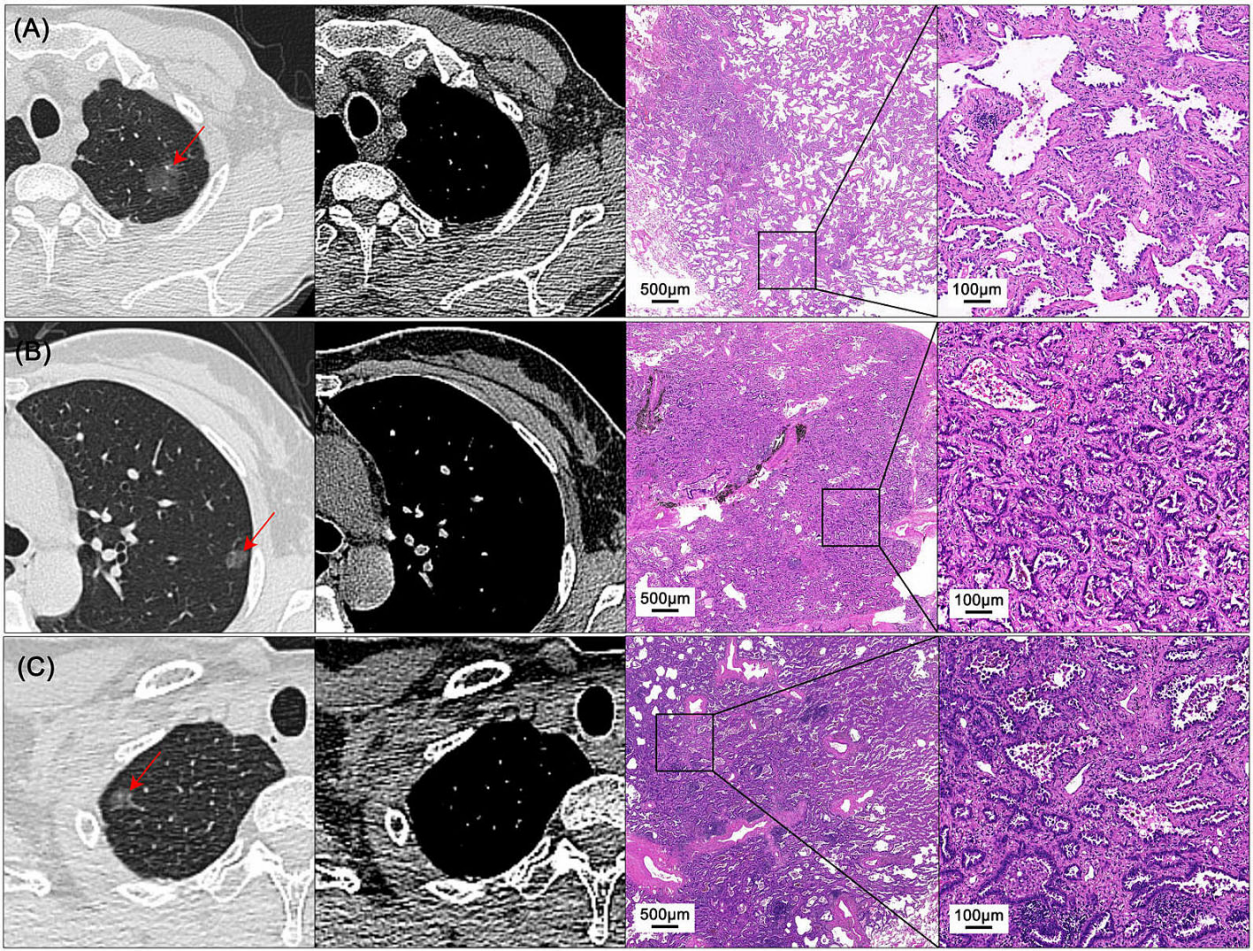
Example of the HRCT images and pathological images for pGGN‐featured IAC. (A) Axial HRCT images and pathological images show a pGGN in the left upper lobe of a 58‐year‐old man. (B) Axial HRCT images for a pGGN in the left lower lobe of a 40‐year‐old woman. (C) Axial HRCT images for a pGGN in the right upper lobe of a 59‐year‐old woman. There are no detectable solid components in all the above HRCT images. HRCT, high‐resolution CT; pGGNs, pure ground glass nodule.

The pathological diagnosis was confirmed based on the 2015 World Health Organization classification of thoracic tumors.^20^ Invasive lung adenocarcinoma was defined as adenocarcinoma with foci of invasion greater than 5 mm. The invasive component includes acinar, papillary, micro‐papillary, and solid architecture, as well as tumor cells infiltrating myofibroblastic stroma. Two pathologists, Liangli Hong and Jinhai Yan, independently examined the section, and final agreements were reached through discussion and consensus. The sub‐histology component was quantitatively recorded at 5% intervals, following the proposal of the International Association for the Study of Lung Cancer (IASLC). The IASLC pathological grade was then determined based on the grading system for IAC published by IASLC in 2020.^15^, ^21^ Examples of the CT images and pathological images for pGGN‐featured IAC are shown in Figure 1.

### 2.3 Surgery

The selection criteria for sublobectomy in this study were as follows: (1) tumors located in the lateral regions of the lung parenchyma; (2) the absence of central bronchial involvement; and (3) the absence of clinical evidence for positive hilar or mediastinal lymph node invasion. Segmentectomy was considered when tumors were confirmed to be confined within the anatomical segmental boundaries, as determined by HRCT, contrast‐enhanced CT, 3D reconstruction, or CT‐guided hook‐wire localization technique. Wedge section was considered for tumors that met the criteria for segmentectomy and were located in the outer third of the lung parenchyma. To ensure a negative margin, a 2‐cm distance from the lesion to the margin should be maintained. If insufficient margins are identified during surgery, frozen section analysis should be performed to confirm a pathologically negative margin. In the event of positive resection margins on frozen section, wedge resection needs to be converted to segmentectomy or lobectomy. For patients who do not meet the criteria for sublobectomy, lobectomy is performed.

We routinely conducted lymph node sampling. Nodal dissection was not required for lobectomy and sublobectomy unless there was a clinical suspicion of lymph node invasion. Further information about the distribution of surgical treatment can be found in Table S1.

### 2.4 Preoperative and follow‐up evaluation

All patients underwent preoperative CT scans and cardiopulmonary function tests. The staging was determined according to the eighth edition of the American Joint Committee on Cancer Staging Manual. All patients were followed up postoperatively at 3‐month to 6‐month intervals after surgery for 5 years and then at 1‐year intervals thereafter. Follow‐up procedures included CT scans and detection of tumor markers.

### 2.5 Statistical analysis

Continuous variables were presented as the mean **±** standard deviation or median (interquartile range), depending on the distribution of the data. Categorical variables were expressed as frequencies and percentages. The comparison of continuous variables was performed using Student's *t*‐test or Mann–Whitney *U* test, while the comparison of categorical variables was conducted using chi‐square or Fisher's exact test.

The primary endpoints of the current study were OS and DFS. OS was defined as the time from surgery until either death or the last follow‐up. DFS was defined as the period from surgery until the appearance of any signs or symptoms of lung cancer.

All tests were two‐sided, and a *p* value below 0.05 was considered to indicate a statistically significant difference. All statistical analyses were performed using SPSS (IBM Corp. Released 2019. IBM SPSS Statistics for Windows. Version 26.0. Armonk, NY: IBM Corp) and R version 4.2.1 (www.r-project.org). R packages including “MatchIt,” “Survival,” and “Survminer” were used.

## 3 RESULTS

### 3.1 Demographic and clinicopathological characteristics

A total of 139 patients with pGGN‐featured IAC were included in this study, with a median age of 60 (IQR 54–69). We combined data from two institutions, taking into account the same distribution and a large difference in sample size (Table S2). The demographic and clinical characteristics are summarized in Table 1. Out of all the patients enrolled, 58 (41.7%) underwent lobectomy, while 81 (58.3%) underwent sublobectomy. Thoracoscopic surgery was performed on all patients, and none of them required conversion to open surgery. Baseline data showed a good balance between the surgery groups. All patients had stage I disease. There were no differences in age (*p* = 0.878), sex (*p* = 0.567), tumor site (*p* = 0.121), tumor size on HRCT (*p* = 0.118), pathological tumor size (*p* = 0.570), diameter of invasive component (*p* = 0.083), or pathological grade (*p* = 0.150). Most of the IAC presented as pGGNs had a tumor size of 0.0–2.0 cm on HRCT (87.8%) and final pathological results (97.1%). No lymph node metastasis was found in any of the patients, indicating a relatively low rate of lymph node metastasis in pGGNs, even when identified as IAC. Nearly 65% of patients who underwent lobectomy also had systematic lymph node dissection. In contrast, only 12.3% of patients who underwent sublobectomy had this procedure (*p* < 0.001). Histopathologic grade 2 tumors accounted for 79.9% of pGGN‐featured IAC, and no grade 3 lesions were identified.

**TABLE 1:** Patient demographics and clinicopathological characteristics.

Variables	Lobectomy (*n* = 57)	Sublobectomy (*n* = 78)	*p*‐value
Age (median [IQR])	60.00 [53, 69]	60.00 [54, 69]	0.834
Sex			0.696
Female	38 (66.7)	47 (61.8)	
Male	19 (33.3)	29 (38.2)	
Tumor site			0.141
Upper	35 (61.4)	54 (69.2)	
Middle	5 (8.8)	1 (1.3)	
Lower	17 (29.8)	23 (29.5)	
Tumor size on HRCT (cm)			0.304
0.0–1.0	11 (19.3)	22 (28.2)	
1.1–2.0	37 (64.9)	49 (62.8)	
2.1–3.0	9 (15.8)	7 (9.0)	
Pathological tumor size (cm)			0.481
0.0–1.0	29 (50.9)	48 (61.5)	
1.1–2.0	26 (45.6)	28 (35.9)	
2.1–3.0	2 (3.5)	2 (2.6)	
Diameter of invasive component (mm, mean ± SD)	8.44 **±** 3.83	7.21 **±** 2.73	0.037
Management of lymph nodes			**<0.001**
Dissection	37 (64.9)	9 (11.5)	
Sampling	20 (35.1)	69 (88.5)	
Pathological grade			0.250
Grade 1	15 (26.3)	13 (16.7)	
Grade 2	42 (73.7)	65 (83.3)	

*Notes*: Data are presented as *n* (%) unless stated. Boldface indicates statistically significant parameters.

Abbreviations: HRCT, high‐resolution CT; IQR, interquartile range; SD, standard deviation.

### 3.2 Recurrence and survival

**FIGURE 2:**
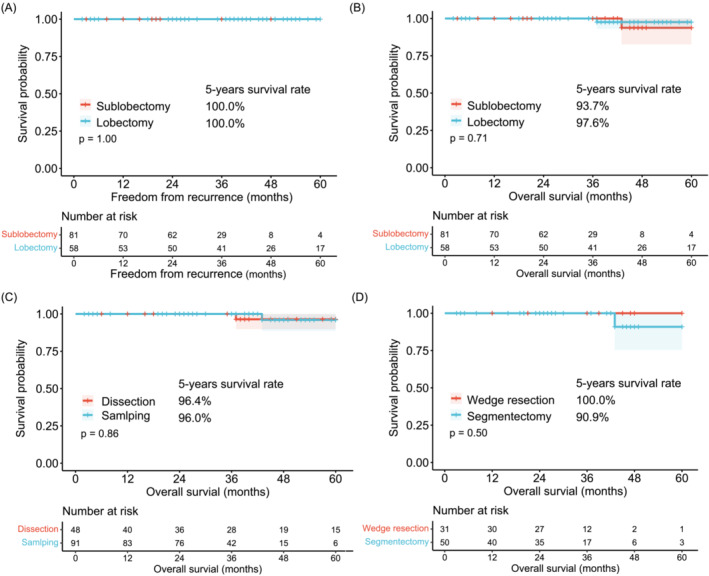
Kaplan–Meier curves comparing different surgical approaches. (A) Kaplan–Meier curves for DFS in the sublobectomy versus lobectomy cohort. (B) Kaplan–Meier curves for OS in the sublobectomy versus lobectomy cohort. (C) Kaplan–Meier curves for OS in the lymph node dissection versus sampling cohort. (D) Kaplan–Meier curves for OS in the wedge resection versus segmentectomy cohort. DFS, disease‐free survival; OS, overall survival.

Overall, the enrolled patients had a 3‐year OS rate of 100% and a 5‐year OS rate of 96.5%. The median follow‐up for the entire cohort was 36 months (IQR, 25–47). No instances of lung cancer recurrence were observed within a 5‐year period (Figure 2A). Throughout the follow‐up period, two patients died, but both deaths were unrelated to cancer. The demographics and clinical characteristics of these patients are summarized in Table S3. There was no statistically significant difference in the 5‐year OS rates between lobectomy and sublobectomy (97.6% vs. 93.7%, *p* = 0.71) according to the Kaplan–Meier survival curves (Figure 2B). Similar results were found in the lymph node dissection versus sampling cohort (96.4% vs. 96.0%, *p* = 0.86) (Figure 2C) and the segmentectomy versus wedge resection cohort (90.9% vs. 100.0%, *p* = 0.50) (Figure 2D).

### 3.3 Stratification analysis

**FIGURE 3:**
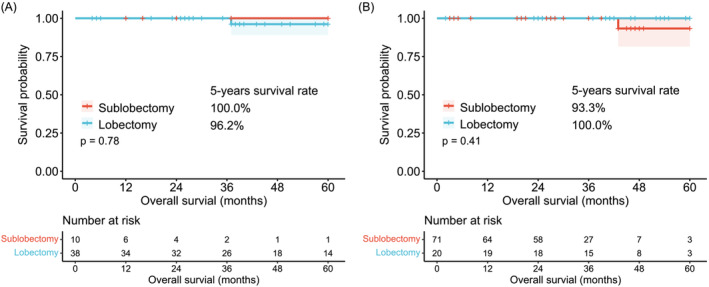
Comparison of sublobectomy and lobectomy stratified by lymph node management. (A) Kaplan–Meier curves for OS in lymph node dissection cohort. (B) Kaplan–Meier curves for OS in the lymph node sampling cohort. OS, overall survival.

**FIGURE 4:**
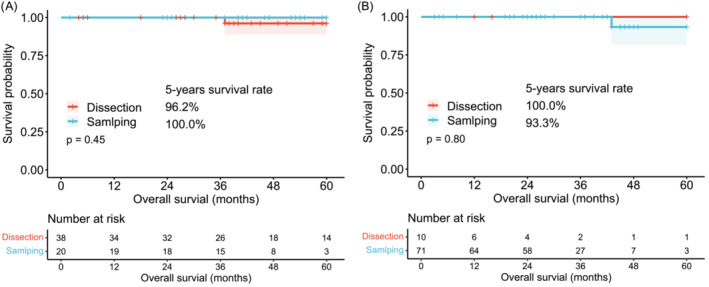
Comparison of lymph node dissection and sampling stratified by surgical methods. (A) Kaplan–Meier curves for OS in the lobectomy cohort. (B) Kaplan–Meier curves for OS in the sublobectomy cohort. OS, overall survival.

As the baseline data revealed a significant difference in the management of lymph nodes and surgical methods, we conducted an additional stratification analysis based on these two variables. No significant differences were found in the 5‐year OS between lobectomy and sublobectomy (100.0% vs. 96.2%, *p* = 0.78) in the lymph dissection cohort (Figure 3A). Similar results were observed in the lymph sampling cohort (93.3% vs. 100.0%, *p* = 0.41) (Figure 3B). Additionally, we did not find a significant difference between lymph node dissection and sampling in the lobectomy cohort (96.2% vs. 100.0%, *p* = 0.45) (Figure 4A) and the sublobectomy cohort (100.0% vs. 93.3%, *p* = 0.80) (Figure 4B).

## 4 DISCUSSION

Lung adenocarcinoma is the most common type of lung cancer, and GGNs are a common radiological feature of this type of cancer.^22^ Previous research has shown a negative correlation between the solid component of GGNs and patients' prognosis.^23^ However, a small number of pGGNs have been identified as IAC.^24^ Due to the poor prognosis of IAC, further assessment of prognosis and optimal surgical approaches for these patients is needed.^21^ In this study, we retrospectively collected and analyzed clinicopathological data from patients with IAC presenting as pGGNs. The results indicated that these patients had an excellent prognosis, with no lymph node metastasis or recurrence. Survival analysis showed no significant difference among different surgical approaches there was no significant difference between lobectomy, segmentectomy, and wedge resection, as well as between lymph node resection and sampling.

The poor prognosis of IAC has been be closely related to the imaging and pathological characteristics.^25^ Sun et al showed that IAC patients with a solid component ratio of less than 50% had a 5‐year DFS and OS of nearly 100%.^22^ Yoshizawa et al studied 514 patients with stage I adenocarcinoma and found that the 5‐year OS for IAC ranged from 61.0% to 75.0% depending on the different pathology subtypes.^26^ Ito et al demonstrated that patients with different invasive tumor sizes and pathological malignant grades had a 5‐year RFS ranging from 67.1% to 96.0%. IASLC grade 3 patients have significantly worse RFS compared to those with grade 1 or grade 2.^25^ In our study, the 5‐year OS for IAC presented as pGGNs after surgery was 96.5%. Although identified as infiltrating lesions, all the pGGN‐featured IAC in the present study were predominantly lepidic, acinar, papillary, and graded as grade 1 or grade 2 according to the IASLC proposal. Therefore, it can be suggested that patients with pGGNs, even those ultimately diagnosed as IAC, exhibit a relatively good prognosis based on the clinical experience and the aforementioned results.

Lobectomy has been the standard surgical treatment for early‐stage NSCLC since 1995.^9^ However, with advancements in imaging and surgical techniques, there has been an increasing attention on the controversy surrounding surgical approaches. Sublobectomy, which removes a more limited portion of the lung tissue, is being advocated by some thoracic surgeons.^11^ This procedure helps preserve lung function and reduces intraoperative blood loss, among other benefits.^27^ In addition, previous studies have revealed that individuals with pGGNs have a relatively high probability of developing second primary GGNs in other lobes, making sublobectomy a more worthwhile consideration.^28^ Sun et al retrospectively investigated 179 patients with clinical stage IA IAC presented as ground glass opacity (GGO)‐dominant nodules and found no statistically significant prognostic difference between lobectomy and sublobectomy.^22^ Qi et al confirmed these results in stage IA1–2 IAC with a consolidation tumor ratio of less than 0.25.^27^ Tsutani et al also showed that there was no difference in outcomes between lobectomy, segmentectomy, and wedge resection, even in patients with GGO‐dominant T1b tumors.^12^ Our study focused on pGGNs and found that the 5‐year OS after sublobectomy did not show significant differences compared to those after lobectomy in patients with pGGN‐featured IAC. Similar results were also found between wedge resection and segmentectomy.

In terms of lymph node management, as the number of patients diagnosed with early‐stage lung cancer increases, recent research agrees that lymph node dissection is unnecessary for them. Matsumura et al conducted a retrospective investigation on 307 patients with cT1aN0M0 NSCLC and found that systematic lymph node dissection may not be necessary for NSCLC smaller than 2 cm with a solid component ratio <75%.^29^ Huang et al also reported similar results.^30^ For pGGNs, Sim et al observed that all the lymph nodes were negative in 64 pGGNs from 48 patients, suggesting that systematic nodal dissection may not be required for these patients.^28^ In our study, we found no lymph node invasion in patients with pGGNs, even though they eventually turned out to have IAC. Our results also indicated that patients who underwent lymph dissection or sampling showed no difference in 5‐year OS, which aligns with previous studies.^31^ Combining the above results with the studies in AIS and MIA,^7^, ^22^ we propose that sublobectomy and lymph node sampling are adequate for patients with pGGNs. However, due to the relatively small sample size and retrospective design, this conclusion should be treated cautiously.

We encountered two deaths in patients with pGGN‐featured IAC: one who underwent lobectomy plus lymph node dissection and the other who underwent sublobectomy plus lymph node sampling. Both patients had R0 resection, but they died within 5 years from causes unrelated to lung cancer. These causes may not have been preventable even if they had undergone standard lobectomy plus lymph node dissection. Therefore, the current results do not support the idea that expanding the detection range with lobectomy or lymph node dissection can improve the prognosis of patients with pGGN‐featured IAC.

There were some limitations to our study. First, as mentioned earlier, the retrospective design of this study is a disadvantage. The surgeon's careful selection of patients who underwent sublobectomy may introduce unmeasured confounding factors, which should be taken into account when interpreting our findings. Second, a primary limitation of this study is the relatively small sample size, particularly for patients with tumor diameters ranging from 2.1 to 3.0 cm. This is due to the rarity of patients with pGGN‐featured IAC. Lastly, our study lacks complete preoperative and postoperative lung function data, which hinders our ability to assess the potential lung function preservation associated with sublobectomy.

In this retrospective study, we found that IAC presenting as pGGNs exhibits low‐grade malignancy and has a favorable prognosis. We did not detect any statistically significant difference in prognosis between lobectomy and sublobectomy, as well as lymph dissection and sampling in patients with pGGN‐featured IAC. Therefore, these patients can be effectively treated with sublobectomy and lymph node sampling.

## AUTHOR CONTRIBUTIONS

All authors had full access to the data in the study and take responsibility for the integrity of the data and the accuracy of the data analysis. *Conceptualization*: Hansheng Wu and Guibin Qiao. *Funding acquisition*: Hansheng Wu and Guibin Qiao. *Formal analysis*: Hansheng Wu and Haijie Xu. *Funding acquisition*: Guibin Qiao. *Investigation*: Haijie Xu, Junhan Wu, Xi Chen, Zihua Lan, Qibin Chen, Jinhai Yan, Shujie Huang, Jianrong Chen, and Xirui Lin. *Methodology*: Junhan Wu, Liangli Hong, and Haijie Xu. *Supervision*: Guibin Qiao and Yong Tang. *Visualization*: Haijie Xu. *Writing—original draft preparation*: Hansheng Wu and Haijie Xu. *Writing—review and editing*: Hansheng Wu, Junhan Wu, Haijie Xu, and Guibin Qiao.

## CONFLICT OF INTEREST STATEMENT

Conflict of interest relevant to this article was not reported.

## ETHICS STATEMENT

All the samples and data were collected after obtaining informed consent from each patient. This research was approved by the Ethics Committee of Guangdong Provincial People's Hospital (GDREC2019687H) and The First Affiliated Hospital of Shantou University Medical College (B‐2022‐149). This study was conducted following the guidelines of the Declaration of Helsinki (as revised in 2013).

## Supporting information


**Table S1.** Distribution of surgical procedures among patients.

**Table S2.** Demographics and clinicopathological characteristics of patients in two institutions.

**Table S3.** Demographics and clinicopathological characteristics for patients who succumbed.

## Data Availability

The data that support the findings of this study are available from the corresponding author upon reasonable request.
