# Supplementary material for: Sublobectomy and lymph node sampling are adequate for patients with invasive lung adenocarcinoma presenting as pure ground glass nodules
Source: Clin Respir J. 2024 May 7;18(5):e13766. doi: 10.1111/crj.13766 (PMC11076303; doi:10.1111/crj.13766)
Supplement: Supplementary file 1 — Table S1. Distribution of surgical procedures among patients. [file CRJ-18-e13766-s001.docx]

**Supplementary Table 1. Distribution of surgical procedures among patients.**

| **Anatomic location** | **Lobectomy** | | |  | **Segmentectomy** | | |  | | **Wedge resection** | | | |
| --- | --- | --- | --- | --- | --- | --- | --- | --- | --- | --- | --- | --- | --- |
|  | **Total** | **dissection** | **sampling** |  | **Total** | **dissection** | **sampling** |  | **Total** | | **dissection** | **sampling** |  |
| **Right upper lobe** | **23** | **15** | **8** |  | **12** | **1** | **11** |  | **13** | | **0** | **13** |  |
| Apical segment |  |  |  |  | 6 | 1 | 5 |  |  | |  |  |  |
| Anterior segment |  |  |  |  | 4 | 0 | 4 |  |  | |  |  |  |
| Posterior segment |  |  |  |  | 1 | 0 | 1 |  |  | |  |  |  |
| Apicoposterior segment |  |  |  |  | 1 | 0 | 1 |  |  | |  |  |  |
| **Right middle lobe** | **5** | **3** | **2** |  | **1** | **1** | **0** |  | **0** | | **0** | **0** |  |
| Medial segment |  |  |  |  | 0 | 0 | 0 |  |  | |  |  |  |
| Lateral segment |  |  |  |  | 1 | 1 | 0 |  |  | |  |  |  |
| **Right lower lobe** | **11** | **9** | **2** |  | **7** | **2** | **5** |  | **6** | | **0** | **6** |  |
| Superior segment |  |  |  |  | 5 | 1 | 4 |  |  | |  |  |  |
| Basilar segment |  |  |  |  | 2 | 1 | 1 |  |  | |  |  |  |
| **Left upper lobe** | **12** | **5** | **7** |  | **22** | **5** | **19** |  | **7** | | **0** | **7** |  |
| Upper division segment |  |  |  |  | 18 | 3 | 15 |  |  | |  |  |  |
| Lingula segment |  |  |  |  | 4 | 0 | 4 |  |  | |  |  |  |
| **Left lower lobe** | **6** | **5** | **1** |  | **6** | **1** | **5** |  | **4** | | **0** | **4** |  |
| Superior segment |  |  |  |  | 4 | 0 | 4 |  |  | |  |  |  |
| Basilar segment |  |  |  |  | 2 | 1 | 1 |  |  | |  |  |  |
