# Supplementary material for: Sublobectomy and lymph node sampling are adequate for patients with invasive lung adenocarcinoma presenting as pure ground glass nodules
Source: Clin Respir J. 2024 May 7;18(5):e13766. doi: 10.1111/crj.13766 (PMC11076303; doi:10.1111/crj.13766)
Supplement: Supplementary file 2 — Table S2. Demographics and clinicopathological characteristics of patients in two institutions. [file CRJ-18-e13766-s003.docx]

**Supplementary Table 2. Demographics and clinicopathological characteristics of patients in two institutions**

| **Variables** | **Guangzhou^†^**  **(n = 121)** | **Shantou^‡^**  **(n = 18)** | ***P*-value** |
| --- | --- | --- | --- |
| **Age (median [IQR])** | 60 [54, 69] | 63 [54, 70] | 0.873 |
| **Sex** |  |  | 0.850 |
| Female | 74 (61.2) | 12 (66.7) |  |
| Male | 47 (38.8) | 6 (33.3) |  |
| **Tumor site** |  |  | 0.189 |
| Upper | 82 (67.8) | 9 (50.0) |  |
| Middle | 35 (28.9) | 7 (38.9) |  |
| Lower | 4 (3.3) | 2 (11.1) |  |
| **Tumor size on HRCT (cm)** |  |  | 0.330 |
| 0.0-1.0 | 31 (25.6) | 3 (16.7) |  |
| 1.1-2.0 | 77 (63.6) | 11 (61.1) |  |
| 2.1-3.0 | 13 (10.7) | 4 (22.2) |  |
| **Pathological tumor size (cm)** |  |  | 0.300 |
| 0.0-1.0 | 71 (58.7) | 8 (44.4) |  |
| 1.1-2.0 | 47 (38.8) | 9 (50.0) |  |
| 2.1-3.0 | 3 (2.5) | 1 (5.6) |  |
| **Diameter of invasive component (mm, mean ± SD)** | 7.72 **±** 3.38 | 7.71 **±** 2.25 | 0.990 |
| **Surgery** |  |  | 0.303 |
| Lobectomy | 53 (43.8) | 5 (27.8) |  |
| Sublobectomy | 68 (56.2) | 13 (72.2) |  |
| **Management of Lymph Nodes** |  |  | 0.081 |
| Dissection | 38 (31.4) | 10 (55.6) |  |
| Sampling | 83 (68.6) | 8 (44.4) |  |
| **Pathological grade** |  |  | 0.161 |
| Grade 1 | 28 (23.1) | 1 (5.6) |  |
| Grade 2 | 93 (76.9) | 17 (94.4) |  |

Data are presented as n (%) unless stated.
† Guangdong Provincial People’s Hospital.

‡ The First Affiliated Hospital of Shantou University Medical College.

IQR: interquartile range, HRCT: high-resolution CT, SD: standard deviation.
