# Supplementary material for: Sublobectomy and lymph node sampling are adequate for patients with invasive lung adenocarcinoma presenting as pure ground glass nodules
Source: Clin Respir J. 2024 May 7;18(5):e13766. doi: 10.1111/crj.13766 (PMC11076303; doi:10.1111/crj.13766)
Supplement: Supplementary file 3 — Table S3. Demographics and clinicopathological characteristics for patients who succumbed. [file CRJ-18-e13766-s002.docx]

**Supplementary Table 3. Demographics and clinicopathological characteristics for patients who succumbed.**

| **No.** | **Surgery** | **Age** | **Sex** | **Tumor size on HRCT (cm)** | **Pathological tumor size (cm)** | **Pathological stage** | **Pathological grade** | **Outcome** | **Causes** |
| --- | --- | --- | --- | --- | --- | --- | --- | --- | --- |
| 1 | sublobectomy + lymph node sampling | 64 | Female | 1.7 | 1.4 | IA2 | Grade 2 | 43 mo, dead | Other reasons |
| 2 | Lobectomy + lymph node dissection | 59 | Male | 2.4 | 1.0 | IA3 | Grade 1 | 37 mo, dead | Other reasons |

HRCT: high-resolution CT.
